# Impact of aerial humidity on seasonal malaria: an ecological study in Zambia

**DOI:** 10.1186/s12936-022-04345-w

**Published:** 2022-11-11

**Authors:** Carolina Duque, Mukuma Lubinda, Japhet Matoba, Caison Sing’anga, Jennifer Stevenson, Timothy Shields, Clive J. Shiff

**Affiliations:** 1 Johns Hopkins School of Medicine, Baltimore, USA; 2 Macha Research Trust, P.O 630166, Choma, Zambia; 3 Johns Hopkins Bloomberg School of Public Health, Baltimore, USA

**Keywords:** Humidity, Plant transpiration, Malaria transmission, Mosquito, Micro-ecology, Zambian rural conditions

## Abstract

**Background:**

Seasonal patterns of malaria cases in many parts of Africa are generally associated with rainfall, yet in the dry seasons, malaria transmission declines but does not always cease. It is important to understand what conditions support these periodic cases. Aerial moisture is thought to be important for mosquito survival and ability to forage, but its role during the dry seasons has not been well studied. During the dry season aerial moisture is minimal, but intermittent periods may arise from the transpiration of peri-domestic trees or from some other sources in the environment. These periods may provide conditions to sustain pockets of mosquitoes that become active and forage, thereby transmitting malaria. In this work, humidity along with other ecological variables that may impact malaria transmission have been examined.

**Methods:**

Negative binomial regression models were used to explore the association between peri-domestic tree humidity and local malaria incidence. This was done using sensitive temperature and humidity loggers in the rural Southern Province of Zambia over three consecutive years. Additional variables including rainfall, temperature and elevation were also explored.

**Results:**

A negative binomial model with no lag was found to best fit the malaria cases for the full year in the evaluated sites of the Southern Province of Zambia. Local tree and granary night-time humidity and temperature were found to be associated with local health centre-reported incidence of malaria, while rainfall and elevation did not significantly contribute to this model. A no lag and one week lag model for the dry season alone also showed a significant effect of humidity, but not temperature, elevation, or rainfall.

**Conclusion:**

The study has shown that throughout the dry season, periodic conditions of sustained humidity occur that may permit foraging by resting mosquitoes, and these periods are associated with increased incidence of malaria cases. These results shed a light on conditions that impact the survival of the common malaria vector species, *Anopheles arabiensis,* in arid seasons and suggests how they emerge to forage when conditions permit.

## Background

In spite of years of research and control efforts, malaria continues to impact much of African society with over 200 million cases per year [1]. The situation has improved in the past 20 years with the use of rapid diagnosis, combination drug treatment, and the rapid implementation of insecticide-treated bed nets and seasonal indoor residual spraying [2]. However, more recently, progress has stalled [2]. In some countries where there are well operated malaria control operations and support from donors such as the Global Fund, prevalence of infection has declined considerably; but there remain some breakthrough cases as well as asymptomatic cases which help perpetuate transmission.

The driving force for malaria transmission is the mosquito vector, as it is the definitive host for *Plasmodium falciparum* and other *Plasmodium* species. In southern Zambia, the main vector species of malaria are members of *Anopheles gambiae* complex, and the most common is *Anopheles arabiensis*, although other species may be involved. The endemicity of malaria is based on the extent and mass of the vector mosquito population [3]. In general, in Africa, when rain falls and there is standing water, mosquito breeding is extensive, and malaria infections become more prevalent. However, in the cooler winter and in the hot dry season surface water becomes sparse and is mostly confined to larger rivers or streams. This results in a reduced opportunity to lay eggs and adult mosquitoes become rare. However, microclimates with periodic increases in humidity during the dry season may present conditions that allow mosquito vectors to seek hosts.

Efforts to detect malaria hot spots have been described, but tended to focus on local breeding sites like streams or ponds [4]. More recently, the importance of surface water or aerial moisture has been studied. In Kenya, a digital elevation model has been used to measure Topographical Wetness Index (TWI), a measure of potential water accumulation due to differences in the terrain [5], and has shown TWI to impact local malaria cases [6]. Malaria hot spots have been described, and have been found to be associated with a range of household and environmental factors, including the prevalence of aquatic habitats [7]. Yet, this may not be the whole story. In the dry season, local streams, ponds and standing water breeding sites are very rare, yet transmission still occurs. There is likely another source or refuge for these mosquitoes.

In dry or cold conditions, various species of *Anopheles* mosquitoes, can adapt to this change in climate, often by entering diapause, a form of dormancy [8]. Work in Mali indicated that *Anopheles coluzzii* has shown evidence of dormancy, but this has not been studied extensively [8]. Another study from Mali also queries dormancy or diapause and suggests mosquito reinvasion from elsewhere may occur [9]. A comprehensive review of diapause and dormancy in mosquitoes states that 11 species of *Anopheles* exhibit dormancy [8]. In addition to diapause and other forms of dormancy, these mosquitoes may also take refuge in moist or covered habitats. In Africa, most trees are not deciduous and thus they hold leaves all year and continue to transpire using ground water. The process of transpiration could produce local microclimates where it is periodically moist and cool, which can allow adult mosquitoes and other Diptera to survive [10]. For example, tsetse flies are known to seek tree buds in the arboreal ecotone when hunting [10]. If these occasional moist periods allow for the local survival or arousal from dormancy, this may result in occasional feeding and periodic transmission of infection [11].

Whether or not there is individual dormancy, mosquitoes do depend on moisture in the air to survive and open water to oviposit. Overall, the literature is still quiescent on the ecological factors that permit mosquito species to forage and survive during the dry seasons and perhaps continue to perpetuate malaria transmission. While it is not clear where adult anophelines rest in the environment during hot and dry conditions, it seems likely that some can survive among the leaves of trees and shrubs [12]. Observations made in Sudan during the dry season, detected *An. gambiae* in wells, soil cracks and other refugia, that were blood fed and viable [13, 14]. Thus, it was hypothesized that under certain conditions of local humidity *Anopheles* mosquitoes might be able to fly, feed, and ultimately transmit malaria. In response, this manuscript examines the potential association between the humidity of microclimates with the incidence of malaria cases.

## Methods

Ethical approval for this study was included in the study entitled “*Malaria transmission and impact of Control Efforts in Southern Africa*” obtained from the Ethics Review Committee of the Tropical Diseases Research Centre (Ndola, Zambia) protocol no. TDRC/ERC/2010/14/11. This permitted collections of mosquitoes in the research area. As the study did not involve data collection on study participants, individual consent was obtained but not required. However, the purpose of the study was explained, and verbal permission was obtained from the household owners to set the loggers in place and requested the house owners to ensure that no one removed or moved the instruments.

### Study area and site selection

**Fig. 1:**
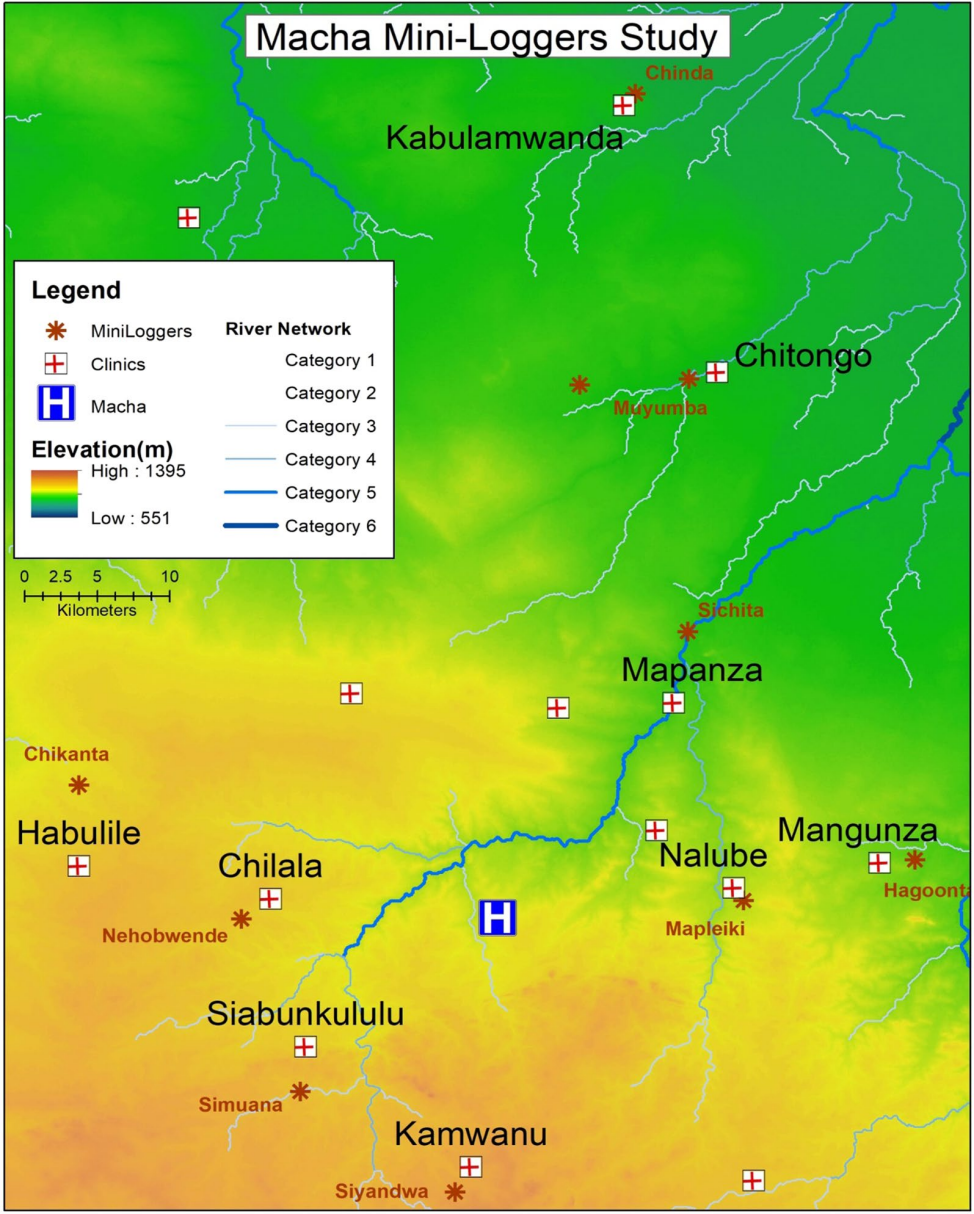
Map of location of clinics and logger location

The work was done in the Southern Province of the Republic of Zambia under the auspices of the Macha Research Trust (MRT). The Macha Hospital, the MRT and the local rural population have established a positive interaction over the past 20 years particularly related to malaria. The countryside in the north is partial flood plain of the Kafue River (Green area in Fig. 1) bisected by several tributaries of the major river. The area is somewhat flat and undulating, with a high water table where there are swamp areas near the tributaries and the local area is approximately 1000 m above sea level. There is a transition to higher ground in the areas marked yellow to orange (Fig. 1), with elevations between 1100 to 1300 m above sea level. The wooded areas are *Brachystegia* and *Terminalia* woodland which includes a variety of tree species.

Much of the area is cleared for agriculture with wooded areas mainly remaining in the drainage areas. Most people live at locations close to their fields. The total area of the study is approximately 5400 km^2^. Nine rural health centres (RHCs) were selected from the 14 RHCs in the ICEMR project under the Macha Hospital catchment area, based on their level of malaria burden between 2013 and 2016. Data on malaria prevalence was obtained from the routine weekly malaria cases by rapid diagnostic test (RDT) recorded in the RHCs. Malaria burden was measured as a rank derived from the total number of RDT positives and test positivity rate. Based on the rankings, five RHCs with the highest malaria burden, and five with the lowest burden were selected for sampling. Macha Mission Hospital was excluded since it serves as a referral facility and thus cases would not be representative of its nearby villages. A list of households with the highest number of malaria cases and those without any cases was obtained from each RHC. This list was then randomized to select the site for data collection. The same was done for low malaria burden RHCs. The selected households were also representative of the population and farming habitat of the Southern Province and are spread out over some several thousand square kilometres. Each single-family domicile was selected from within approximately 5 km from a rural health centre (Fig. 1).

Each site was located on Google Earth and images were obtained to assess the tree cover and location of the sample. Locations were examined for consistency of size and occupation. The homestead usually consisted of several single-room huts, circular or square mud or brick wall houses with thatch roof. There was also open air but roofed recreation/cooking areas and several trees in the location. These provided shade and were used day and evening for meals and convocation. Each location had an outdoor granary to store maize and other crop harvests. Other structures were latrines and bathing enclosures. In all locations, there was no evidence of anopheline oviposition sites particularly during the hot dry season. Domestic water was obtained from wells or natural streams usually several hundred metres from the homestead. Culicines could easily inhabit the wells, but these were not suitable habitat for larval anopheline mosquitoes.

### Local weather data

**Fig. 2:**
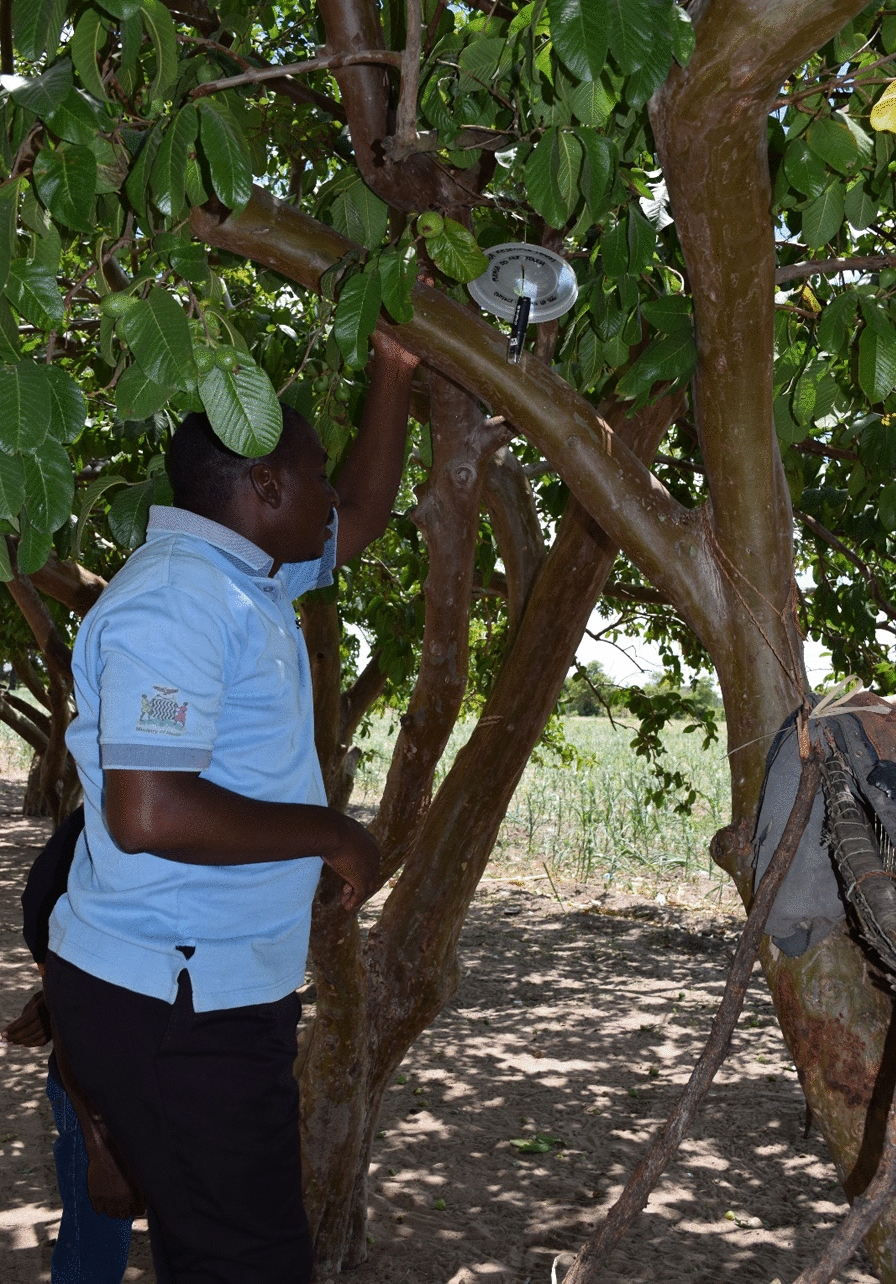
Photo showing the positioning of the logger among the trees. Note the shield to protect the instrument from sun and rainfall

Battery-operated loggers (LASCAR Electronics EasyLog EL-USB-2 (www.lascarelectronics.com) were placed in a tree in the vicinity of the homestead, and on the outside of the granary at each of the localities. Tree loggers were placed under a cluster of leaves (Fig. 2), and the tree species to which they were attached was identified, where possible (Table 1). There was no intrusion of the homestead or requirement of the homeowner except that they knew what was being measured and agreed to ensure the safety of the measuring apparatus. In the 3 years of recording only one logger was lost and was subsequently replaced.

**Table 1:** Sites of logger location [35]

Logger site	Tree scientific name	Coordinates	Elevation (m)	Adjacent Rural Health Centre
Chikanta	*Brachystegia boehmii*	448,355/8197121	1149	Habulile
Nehobwende	*Piliostigma thonningii*	460,068/8187527	1192	Chilala
Simuwana	*Brachystegia spiciformis*	464,128/8175165	1210	Siabankulu
Chiinda	*Faidherbia albida*	487,251/8246618	1008*	Kabulamwanda
Muyumba	many trees and shrubs	490,950/8226205	1013*	Chitonga
Hagoonta	*Brachystegia spiciformis*	506,339/8191770	1069*	Mangu’unza
Mapleiki	*Terminalia sericea*	494,717/8188852	1121	Nalube
Siyandwa	*Strychnos spinosa*	464,128/8175165	1268	Kamwanu
Sichita	*Pseudolachnostylis maprounefolia*	490,858/8208122	1047*	Mapanza

* Located on or adjacent to the Kafue River flood plain

The loggers recorded temperature, relative humidity, and dew point every hour from September 2017 to December 2019. The devices were suspended among leaves with a small plastic disc to protect the logger from excessive rain and direct sunlight, as the objective was to measure aerial conditions, not extreme temperature (Fig. 2). The granary sites were open and unshaded. During the dry season, the loggers were examined, tested and data downloaded monthly. During the other seasons, loggers were visited bimonthly. Non-functioning loggers and batteries were replaced if necessary.

Rainfall was measured at a single location at the Macha Research Trust location using a HOBO Meteorological Station and accessed weekly. Rainfall at Macha was assumed to be representative of the overall region, as the wet season is derived from the continental inter-tropical convergence zone which covers large areas in Southern Africa.

### Mosquito sampling

Standard Centre for Disease Control (CDC) light traps were used for collecting mosquitoes at the nine households in the study. The CDC light traps were set overnight from 6:00 p.m. to 6:00 a.m. The light traps were placed indoors next to a sleeping space and outdoors in either a cattle or goat pen, if available at the household. The mosquito traps were set overnight once every month from August to November, and at opportune times the rest of the months. Due to logistical challenges, mosquito collections for 2018 were only done in January, February, March, and December. These collections were useful for assessing the various species of anophelines collected over the period of study.

Mosquitoes collected from the light traps were first morphologically identified by entomologists at MRT and then by polymerase chain reaction (PCR) for specific individual mosquito species identification, as previously described [15].

### Malaria incidence

Malaria incidence data were collected from the Rural Health Centre closest to each of the nine villages (Fig. 1). Each health centre/clinic has been recording weekly positive malaria rapid diagnostic test results since 2008. These clinics are staffed by government nursing personnel and are the main source of health service for the rural population. These health centres participate in an ICEMR programme and are monitored regularly by research personnel from Macha Research Trust. The clinic personnel are alert to malaria and are required by the Zambian Ministry of Health to administer a rapid diagnostic test if they suspect malaria [16]. Every week, each clinic sends case data relating to malaria and other infectious diseases to the Macha Research Trust personnel, and these data were routinely checked and found to be reliable [17]. This includes the total number of RDT units used during the week, and the number and age of all positive results.

The clinic staff also maintain medical case histories of all the people they serve and can thereby estimate the population attending the clinic. This yearly population estimate was used to calculate the malaria incidence rate since the populations are stable with very little emigration or immigration. Incidence per 100,000 person-weeks was calculated based on these yearly population estimates. If a population estimate was not available for a particular year, the previous year’s population was used.

### Data analysis

#### Analysis of weather trends

Humidity and temperature were analysed between the night-time hours of 6:00 p.m and 6:00 a.m., as this is when the temperature is lowest and mosquitoes, being nocturnal feeders, are most active. For the 2.5 years of the study, weekly and monthly nighttime counts of three-hour intervals above 50% relative humidity, were calculated. The choice of a three-hour interval of humidity was selected somewhat arbitrarily assuming that such a period would likely be sufficient for the mosquito to attempt to forage. The 50% humidity threshold was selected based on reviewed literature [11, 18, 19] and observations in the MRT semi-field system where mosquitoes were only caught in light traps when relative humidity was at least around 50%. The humidity counts were then compared between the tree and granary sites of each village using a paired Wilcoxon Ranked Sum Test and between all the villages using Kruskal–Wallis Test. The humidity and temperature data recorded in trees were correlated to those found in the granaries by the Spearman rank correlation coefficient. Analysis was run on R statistical software, version 4.0.4 [20].

Monthly nighttime humidity counts, and temperatures were averaged across the village sites, and malaria case incidence per 100,000 person months was pooled across all nine health centres. Rainfall, measured at Macha, was considered representative of the overall region. These monthly values were adjusted to account for missing data and differences in the number of days per month, and then plotted to explore seasonal trends.

#### Negative binomial regression analysis

A series of negative binomial regression models were employed to explore the association between environmental factors and malaria incidence. A negative binomial model was chosen because the malaria case data were over-dispersed. For each model, the response variable was weekly malaria incidence, and the fixed effects were weekly nighttime counts of RH greater than 50%, average nighttime temperatures, rainfall, elevation, and year. Village was added as a random effect and a population offset was used to account for changes in village population year-to-year. Separate models were built for the whole year and for the dry season (June through October). While May is also a dry month, June was used as a starting point to prevent carry over of cases from the rainy season. Next, to assess the temporal association between malaria incidence and the fixed variables, given the malaria incubation period of approximately 4–14 days, the models were built with zero, one-week or two-week lags in malaria cases (Table 2). Next, backward stepwise selection of the variables was done by removing variables one at a time, until the lowest Akaike Information Criterion (AIC) was achieved. Finally, the AIC was also used to determine the best model for the lag between malaria incidence and the fixed variables. This process was repeated for the granary data as well. Missing data were excluded from the models with 400 data points missing in the whole year tree model, 592 in the granary whole year model, 144 in the tree dry season model, and 240 in the granary dry season model. Analysis was run on R statistical software, version 4.0.4 [20].

**Table 2:** Selection of negative binomial models and variables

	Offset	AIC	P value				
			Humidity	Temperature	Year	Rain	Elevation
Full Whole Year Tree Models	No lag	1411.45	**1.06 E−17**	**1.72 E−03**	**1.61 E−04**	0.931	0.530
	1 week lag	1459.25	**2.70 E−16**	**3.96 E−09**	**4.28 E−05**	0.687	0.427
	2 week lag	1498.52	**1.70 E−14**	**3.22 E−12**	**2.68 E−04**	0.308	0.275
Full Dry season Tree Models	no lag	329.5862	**1.29 E−04**	0.231	0.473	0.304	0.324
	1 week lag	294.8394	**4.05 E−04**	0.583	0.614	0.185	0.472
	2 week lag	287.3746	0.697	0.848	0.795	0.842	0.172

Negative binomial models with all variables included for full year and dry season, based on tree or granary data. Each model is shown with offsets of zero 1-, or 2-week lags. Lower akaike information criterion (AIC) indicates better fitting model. P-values are reported for each variable in the full model

## Results

### Evaluation of weather trends and malaria incidence

Using previous insectary observations that mosquitoes can become active and seek out bloodmeals after three consecutive hours with relative humidity above 50%, household hourly logger data was used to establish weekly and monthly counts of nighttime 3-h intervals above 50% RH. The database derived from these 2.5-years of measurements amounted to 319,855 records. During this same period, reports of malaria cases were obtained weekly from nine nearby clinics, detecting a total of 554 cases. These clinics combined served an estimated population of 104,712 in 2017, 107,009 in 2018 and 112,138 in 2019.

**Fig. 3:**
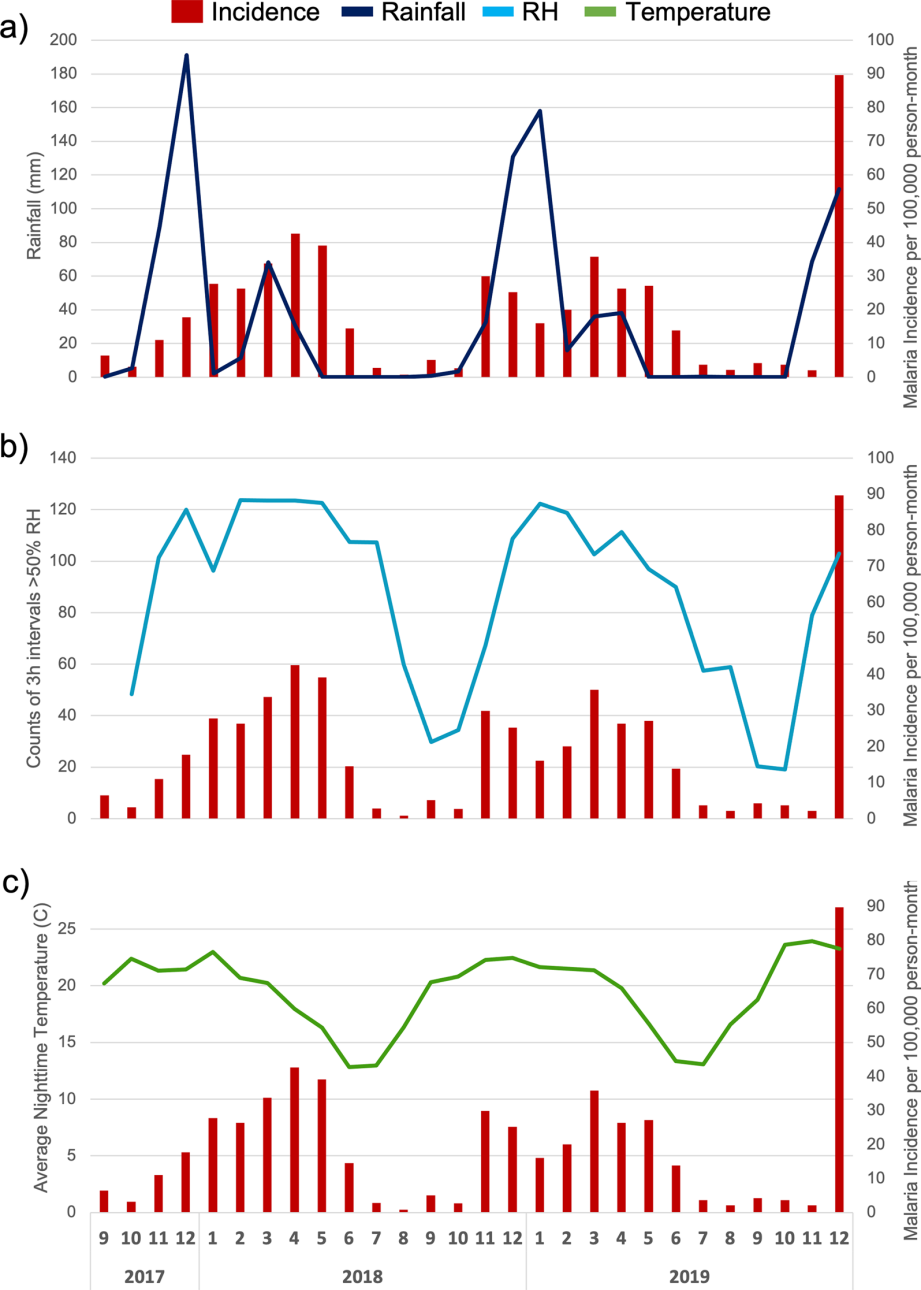
Malaria incidence per 100,000 person-months (red) pooled across all 9 villages. **a** Monthly rainfall (dark blue) at Macha. **b** Monthly counts of night-time 3-h intervals above 50% relative humidity (light blue) averaged across all 9 villages. **c** Monthly nighttime temperatures (green) averaged across all 9 villages. All monthly values were adjusted for different number of days per month and for missing data

The monthly malaria incidence, nighttime humidity counts, and nighttime temperature were averaged across the sampled sites to determine the temporal trends for the region as a whole (Fig. 3). Rain was measured at a single site but was considered representative of the region. Rain primarily fell in the months of November (M = 63.5 mm, SD = 28.5) and December (M = 144 mm, SD = 41.4) with some more sporadic rain through to April. From the months of May to October, there was virtually no rain, with 3 mm in 2018 and 0.4 mm in 2019. Nighttime temperatures at the tree sites were highest during the rainy season of November to April with an average of 21.5 °C (SD 3.96). Temperatures began to drop in April and reach lows during June and July (M = 13.1 °C, SD = 4.94), before slowly rising again in August. The average nighttime temperature during the dry season of May to October was 18.9 °C (SD = 6.14). The average nighttime temperatures of the trees were strongly correlated with those of the granaries (R = 0.98, P < 0.001), but were overall significantly colder in the trees than the granaries (P < 0.001).

During the rainy season, nighttime relative humidity of tree sites was consistently above 50%, with average monthly counts of relative humidity above 50% for 3 h of 101.1 (SD 16.4) which corresponds to 25 full nights a month with relative humidity above 50% for 3 h. Notably, elevated humidity levels extended beyond the rainy season into June before rapidly dropping off. From May to June of the dry season, the average monthly counts of relative humidity above 50% for 3 h was 102.6 (SD = 15.0) while from July to October it was 46.5 (SD = 27.5). This prolonged increase of humidity into the dry months may be the result of standing pools of water that remain from the previous rainfalls although this is unlikely due to overall aridity and/or from increased plant transpiration. Indeed, while the tree and granary humidity readings above 50% relative humidity for 3 consecutive hours were correlated (R = 0.84, P = 2.2 × 10^–16^), the tree sites were found to have significantly more humidity readings above 50% RH for 3 consecutive hours compared to granary(P < 0.001). There was no significant difference in humidity readings from village to village (H(8) = 8.802. P = 0.036.

Malaria incidence was also notably highest in the rainy season with an average monthly malaria incidence of 28.88 cases per 100,000 person-months (SD 20.36), but was also high, at 23.64 cases per 100,000 person-months (SD = 10.87), during the early dry season of May and June when the rain had stopped but humidity was still elevated. Once humidity declined in July through October, the incidence dropped to 3.46 cases per 100,000 person-months (SD = 1.55) (Fig. 3).

### Modelling the role of weather variables on malaria incidence

**Fig. 4:**
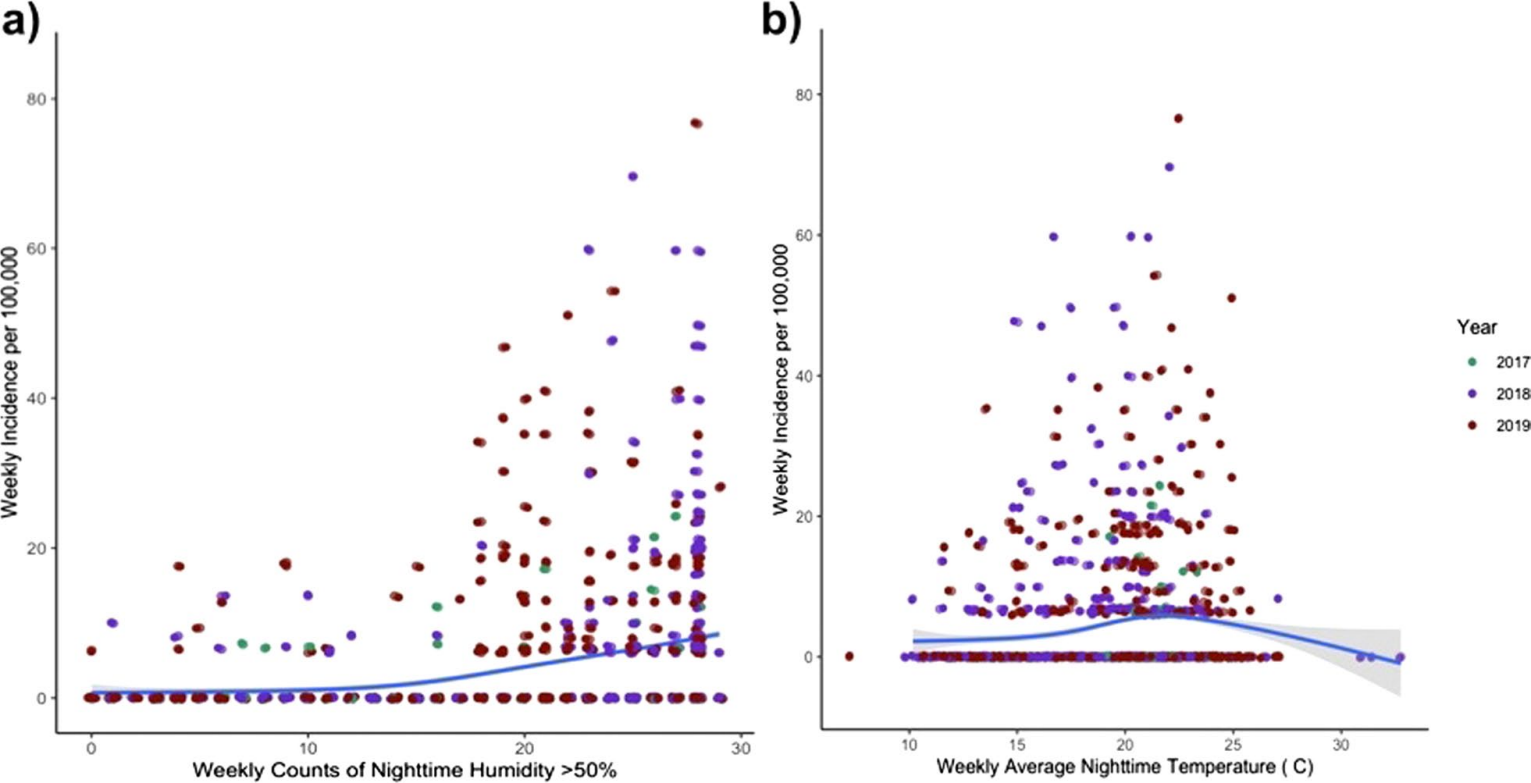
**a** Weekly malaria incidence per 100,000 person-weeks for the full year by the weekly count of night-time 3-h intervals with greater than 50% relative humidity in the trees, and **b** Weekly malaria incidence per 100,000 person-weeks for the full year by the average weekly nighttime temperature in the trees. Each point represents a single week for a given village and corresponding health centre. The blue line represents the full year tree model predictors

To investigate further the potential role of humidity on malaria incidence, a series of negative binomial regression models were developed using whole year local weather readings from tree loggers. All models used weekly malaria incidence as the response variable, and evaluated the role of sustained humidity, temperature, elevation, and year as fixed variables in the model. From the full model containing all these variables, backward selection, based on AIC, identified weekly nighttime counts of sustained relative humidity, average nighttime temperature, year and rainfall as important variables in the model. Elevation was not included in the final model due to worsening the strength of the model, based on AIC, and a non-significant (P > 0.05) effect in the model. Notably, rainfall did not have a significant effect in the model (P > 0.05), but its removal resulted in a weaker model, based on AIC, so it was kept in the final model. Humidity, temperature and year were all found to contribute significantly (P < 0.01) to the whole year model (Table 3). In addition, the strongest model, based on AIC, was the final whole year with no temporal lag between the environmental variables and the malaria case data (Table 3). In this model, if all other variables are held constant, for every additional count of a three-hour interval with RH above 50% in trees, the weekly incidence of malaria increases by 11.1% (IRR = 1.111, 95%CI = 1.084–1.138), and for every degree increase in temperature in trees, the weekly incidence increased by 7.4% (IRR = 1.074, 95% Cl = 1.026–1.145). This relationship is shown graphically in Fig. 4. To evaluate, the more widespread effects of humidity, a similar model was created using the same parameters as the final whole year tree model, but with the humidity and temperature data recorded in granary sites. In the granaries, for every unit increase in counts of 3-h intervals with humidity above 50%, the weekly incidence of malaria increased by 5.3% (IRR = 1.053, 95%CI = 1.023–1.077), and for every degree increase in temperature, malaria incidence increased by 9.8% (IRR = 1.098, 95% CI = 1.041–1.159). These findings overall align with what is seen in the tree models, yet granary humidity seems to play less of a role in malaria incidence compared to trees given that its effect size, based on IRR, is nearly half that of the tree humidity.

**Table 3:** Final negative binomial models derived from backward selection of full model variables

	Model	AIC	P value					IRR		
			Humidity	Temperature	Year	Elevation	Rain	Humidity	Temperature	Rain
Final Whole Year Tree Models	No lag	1409.8	**1.22 E−17**	**2.03 E−03**	**1.62 E−04**	NA	0.891	1.111	1.074	1.000
	1 week lag	1457.9	**3.23 E−16**	**5.20 E−09**	**4.35 E−05**	NA	0.664	1.097	1.143	1.000
	2 week lag	1498.5	**1.70 E−14**	**3.22 E−12**	**2.68 E−04**	0.2750	0.308	1.083	1.180	0.995
Final Dry Season Tree Models	No lag	324.59	**4.35 E−05**	NA	NA	NA	0.349	1.102	NA	0.499
	1 week lag	290.8	**7.77 E−04**	NA	0.690	NA	0.127	1.093	NA	1.278
	2 week lag	281.45	0.522	NA	NA	NA	0.875	1.016	NA	1.031

This table includes the final models for whole year or dry season, based on tree or granary data, and zero-, 1- and 2-week lags. Lower akaike information criterion (AIC) indicates better fitting model. P-values for each variable included in the model are shown, and in bold are significant values. If a variable was not included in the final model it is listed as NA. The Incidence Rate Ratio (IRR), indicates the ratio by which malaria incidence increases for every unit increase of humidity or temperature, as other variables are held constant

**Fig. 5:**
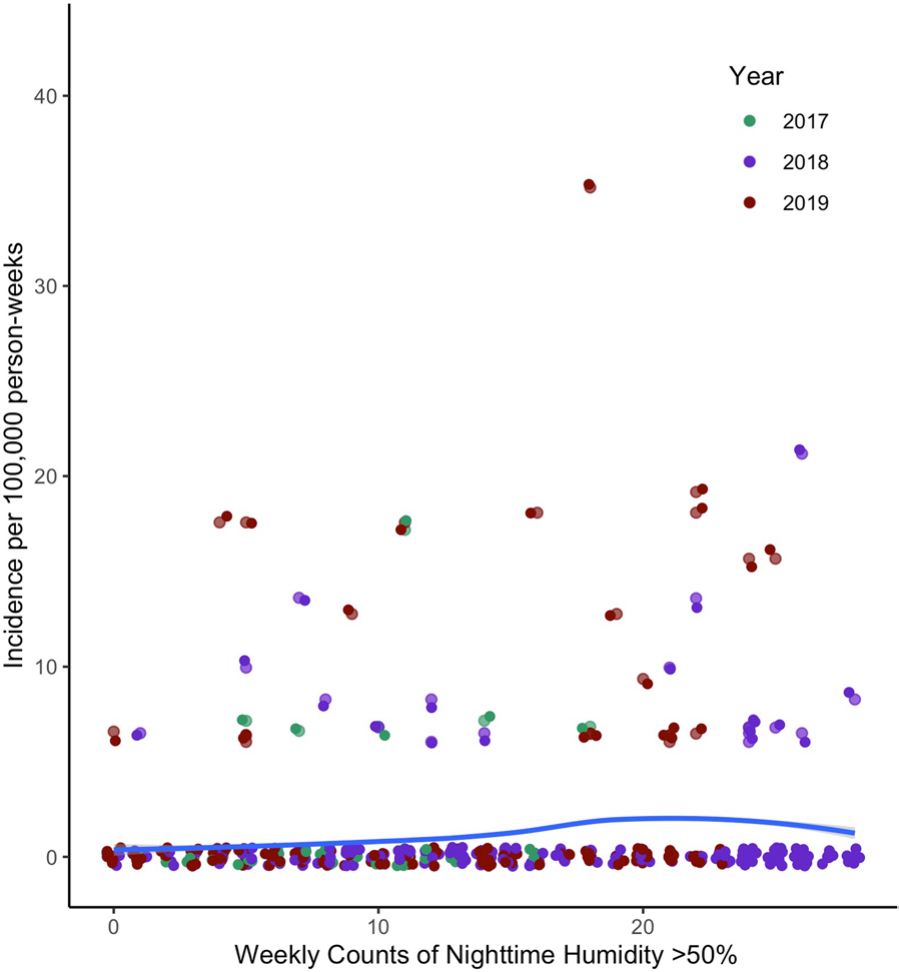
Weekly malaria incidence per 100,000 person-weeks for the dry season (June to October) by the weekly count of night-time 3-h intervals with greater than 50% relative humidity in the trees. Each point represents a single week for a given village and corresponding health centre. The blue line represents the full year tree model predictors

Next, given the interest in better understanding malaria incidence during the dry season (June to October), a series of models were created for this subset of the year. This was done to investigate whether humidity continues to play a role, even in the absence of rainfall. Based on AIC, the final model corresponded to a two-week lag, but in this model no variables were considered significant (Table 3). The next best fitting model used a one-week lag. Backward selection identified only humidity, rainfall and year as important variables to include in this final model. A zero-week lag model that more closely matched the whole year model, also identified humidity and rainfall as important variables to include. For both these models, while rainfall was considered important to the strength of the model by AIC, it was not statistically significant by P-value (P > 0.05). Rainfall during the dry season was only detected in three weeks over the three years of the study and totaled only 8.6 mm. Conversely, local tree humidity was strongly significant in the zero and 1-week lag models (Table 3). The 2-week lag model, which had the best AIC, did not show significance for any of the variables evaluated. This may be due either to a true lack of effect or the fact that there are so few malaria cases in the dry season, especially when shifting 2 weeks further into the dry season, that the distribution of the negative binomial becomes so narrow that it is difficult to fit the model. In the 1-week lag model, which was the next best fitting model, for every additional count of a three-hour interval with RH above 50% in trees, the weekly incidence of malaria increases by 9.3% (IRR = 1.093, 95%CI = 1.040–1.153). This weakly positive association between night-time tree humidity counts and malaria incidence is shown graphically in Fig. 5. Very similar results are found when the same model was created using granary data, where for every additional count of a three-hour interval with RH above 50% in granaries, the weekly incidence of malaria increases by 10.2% (IRR = 1.102, 95%CI = 1.031–1.180). These findings suggests that, although malaria cases are low during the dry season, there appears to be an association with humidity. Thus, sustained humidity may play a role in the periodic transmission of malaria during the dry season of southern Zambia.

### Mosquito collections

To determine the species of anopheline mosquitos in the villages being studied, CDC light traps that were intermittently placed across the nine sites throughout the study period. In the 294 traps that were placed, a total of 2605 mosquitos were caught. Of those, 557 were morphologically determined to be female anopheline mosquitos. PCR was able to be performed on 266 of these, and 200 were identified. Of those identified by PCR, 62 (31.0%) were *An. arabiensis*, 89 (44.5%) *Anopheles squamosus*, 30 (15.0%) *Anopheles quadriannulatus,* 18 (9.0%) *Anopheles rufipes*, and 1 (0.5%) *Anopheles pretoriensis*.

Of all the traps placed, 101 were placed in the dry season of June–October catching a total of 126 mosquitos. 24 of these were determined to be female anopheline mosquitos, of which 8 were speciated by PCR. These were identified as 3 *An. rufipes*, 2 *An. arabiensis*, 2 *An. quadriannulatus*, and 1 *An. squamosus.* Although, the collections were sporadic due to study limitations, these findings still provide valuable information regarding the species of mosquitoes in the region.

## Discussion

In several recent publications on malaria epidemiology relative humidity is mentioned, but often the measurements have been underestimated and often quoted from average numbers or based on meteorological data from a collecting station [11, 21]. Even recently previous work in Zambia attempted to measure local relative humidity around villages near the Zambezi River using satellite information; it was difficult to transfer dew point measurements by the Landsat satellite to local relative humidity in a meaningful manner [22]. Obviously more precise meteorological measurements are needed. The small LASCAR temperature and humidity loggers used in this study have enabled collection of data from microhabitats and hourly measurements throughout the 24-h cycle for three consecutive years. In 2018, a group from Chennai in India used similar loggers to assess microclimate variables of the ambient environment to study the extrinsic incubation period of *Plasmodium vivax* and *P. falciparum* in an urban setting [23].

The study reported here, has taken a broad climatic view covering large tracts of land and attempted to consolidate ecological parameters into a large territorial ecosystem. This study measured a series of ecological features that impact the transmission of malaria through their effects on the natural ecology of the vector mosquito. For temperature, this study shows that it has an important role on the incidence of malaria when looking at the full year. The models in this study show that, when holding all variables constant, malaria incidence increases as temperature increases. When all variables are included, the model predictors show that beyond a certain temperature, malaria incidence again begins to fall. This may be due to the fact that as temperature increases, humidity falls. Recent literature is attempting to ascertain temperature limits. Shapiro et al. [24] used *Anopheles stephensi* and *P. falciparum* with selected variations of temperature from 21-34C and measured half-life periods, however they did not consider daily fluctuations as would happen in nature [24]. Lunde et al. [25] examined six different survival models, but again did not address the issue of variability [26]. Other field-based modelling studies, that do account for daily variability, have shown similar effects of temperature on malaria incidence [27–30]. Interestingly, for the dry season model, the data indicate that temperature does not have a significant effect.

The only variable that was found to be significant in both the full year and the dry season was sustained relative humidity, the main objective of this study. These models indicate that, as the intervals of sustained relative humidity above 50% increased, there was an increase in malaria incidence. Interestingly, this effect was not significant when looking at the dry season of June–October with a two-week lag. While it may be possible that humidity truly does not have an effect for this period, this lack of significance may also be due to the fact that the case load is so low in the area during this period that it becomes statistically challenging to detect any effect due to the limited range of incidence.

Determining where this humidity comes from was not the objective of this study, but it is an important question to consider. In the hot dry season in South Central Africa, there is little moisture in the environment. There is no rainfall, no standing pools of water and no isolated mists observed by local people. Large rivers do remain, but these are located far from the villages used in this study. It is possible that the humidity produced by these rivers may be brought to the villages by local wind movement. Alternatively, this humidity may be derived from tree transpiration. This study showed that trees have more sustained humidity readings above 50% than the granaries in the same village. These humidity readings are still correlated, thus is it possible that trees produce enough humidity to diffuse throughout the local compound. Overall, further studies are needed to better establish the source of the humidity.

*Anopheles* mosquitoes have clearly adapted to the variations in climate throughout Africa. As has been shown here, and as others have shown elsewhere [13, 31], these mosquitoes can survive and forage all year long [18]. The study results here suggests that this survival and/or ability to forage is dependent on humidity and temperature. During the dry seasons this association was weaker but further studies in areas with greater malaria caseloads may be able to better establish this association. Local persistent aerial moisture will also depend on movement of air, storms, and stillness, which were not measured in this study. This will be an important variable to evaluate in further studies.

Research carried out in the Sahel and other countries on members of the *An. gambiae* complex have been studied throughout dry seasons to investigate diapause, dormancy, migration and effects on the mosquito populations [21, 32–34]. Population studies involving genetic markers in *An. arabiensis* in Senegal showed little change in the genetic profile across the area from dry to rain season and back [19]. The authors’ consensus was that local survivors were derived from a permanent population deme spread over large areas that fluctuates seasonally. The results of this study tends to support these results. Humidity can assist in the survival of pockets of females which are present in the area and when the rains occur in November this enables the mosquito population to expand rapidly. This is likely to occur over much of the African continent and needs to be considered wherever malaria control is practiced.

### Limitations

The work reported here covers data collected from nine operational loggers set in growing trees, near houses where people sleep, which more closely represents the ecological features of the resting mosquito. However, as with most field-based studies there are limitations to this study. These include missing data due to broken or displaced loggers, and logistical circumstances limiting mosquito collection and the period of data collection to 2.5 years. In addition, while many climatic variables were able to be included in these models, some, such as wind, could not be measured. Furthermore, some of the infections may have been missed since the diagnoses were made using malaria rapid diagnostic tests, which are less sensitive than blood smear examination, and because many individuals are likely never tested because they do not seek care for asymptomatic or mild cases. These challenges particularly affected the ability to fit a model to the dry season, but important trends were still found.

## Conclusions

This study aims to show the importance of local humidity in supporting mosquito foraging and malaria transmission in agricultural areas in central southern Africa. These conditions appear to help maintain local transmission of malaria and to also provide conditions that enable local foci of vector species to survive and expand when rains occur. These factors will be essential to consider when establishing proper malaria control efforts in these areas.

## Data Availability

The entire data set is on file both in the authors files in Baltimore (cshiff1@jhu.edu) or Zambia (mukuma.lubinda@macharesearch.org). All data will be available on request.
